# Increasing shareholder focus: the repercussions of the 2015 corporate governance reform in Japan

**DOI:** 10.1007/s10997-021-09619-0

**Published:** 2021-12-23

**Authors:** Paweł Mielcarz, Dmytro Osiichuk, Karolina Puławska

**Affiliations:** 1 Department of Finance, Kozminski University, 03-301, 57/59 Jagiellonska St., Warsaw, Poland; 2 Department of Accounting, Kozminski University, Warsaw, Poland

**Keywords:** Corporate governance, Board independence, Profitability, G30

## Abstract

The corporate governance reform promulgated in 2015 in Japan has contributed to a substantial increase of board independence and a reduction of average board tenure. Our empirical analysis covering 3405 public companies demonstrates that reinvigorated corporate oversight and an increasing post-reform shift towards prioritization of shareholder value have led to a persistent increase of corporate profitability, asset productivity, dividend payouts, acquisitions’ value, and valuation multiples. We also document a significant increase of sensitivity of executives’ and directors’ compensations to the dynamics of firms’ bottom lines. The positive changes are the most pronounced within companies where independent directors constitute a majority on the board. The most notable drawbacks of the reform are a significant reduction in net employment creation and in employee turnover within the largest companies. These might be a possible reason for the documented improvement in corporate performance. The number of part-time employees has also seen a significant increase. While being prima facie focused on reinvigorating the private sector, the corporate governance reform may implicitly undermine the established social contract based on job security. Therefore, our study is important from the perspective of sustainable development of the corporate sector as it demonstrates that while concentrating on improving corporate governance, it is also necessary to consider the business’ social responsibility.

## Introduction

On March 5, 2015, Japan's Financial Services Agency published Japan's Corporate Governance Code (Code), which was enacted in June 2015 (Code, 2015). One of the major changes to the architecture of corporate governance mechanisms was a formal imposition of a requirement for firms listed on Sects. 1 and 2 of the Tokyo Stock Exchange to have at least two independent supervisory board members. The principal goal of the Code was to contribute to the broader Japan Revitalization Strategy (Spiegel, 2018) by increasing the top-down pressure on corporate executives to implement operational restructuring aimed at increasing business efficiency. In turn, the strategy adopted in 2013 is pursuing the goal of increasing the role of institutional investors in performing their stewardship role and navigating firms towards faster growth (Code, 2015).

In June 2021, the Code was revised. One of the aims of new version of the Code was to increase the Japanese corporations’ awareness of sustainability issues (environment, social and governance factors, ESG) (Code, 2021). The revised version of the Code states that sustainable development is an important management issue from the perspective of increasing mid- to long-term corporate value. One of the concerns stressed in the Code is the boards’ responsibility to ensure fair and appropriate treatment of the workforce, including caring for their health and working environment (Code, 2021). However, the Covid-19 pandemic revealed important deficiencies in the corporate policies aimed at ameliorating health and safety standards as well as overall working conditions within the Japanese corporate sector.

The emergence of ambitious projects of corporate governance reform was fueled by the wide-spread belief that Japan needed a more dynamic and better performing private corporate sector to respond to the challenges posed by dire demographic situation, slow economic growth, and gradual deterioration of the country’s fiscal balance (Hirakata et al., 2016; Ogawa et al., 2017; Toshiyuki et al., 2010).

Corporate oversight reinvigorated by the reform was supposed to shift the focus of corporate performance management towards prioritization of shareholders’ interests. For years, Japanese companies have been underperforming their US and European peers in terms of profitability (Fu & Ogura, 2019), efficiency of resource deployment, and shareholder returns (Khuu et al., 2016). The common belief is that part of the reason for inferior performance scorecard is the rigidity and duality of the Japanese labor market, which offers employees unparalleled job security (Aoyagi & Ganelli, 2015; Esteban-Pretel et al., 2017). The pre-existing institutional and informal limitations imposed on labor relations have been shown to reduce the mobility of workforce and re-deployment of resources towards more productive uses (Nakamura et al., 2019). The latter, in turn, constrained the medium- and long-term growth of the total factor productivity.

Attention has also been drawn to the perceived flaws of the corporate governance system prevailing within Japanese companies. To start with, firms are prioritizing internal promotions over external talent acquisition (Ariga et al., 1992; Kawagushi, 2015), which significantly limits the pool of available workforce to recruit senior officers. Secondly, supervisory boards have also been staffed with corporate insiders, which reinforced internal cohesion and facilitated dialogues between corporate stakeholders, but did so at the expense of efficiency, resilience, and shareholder returns (Baran & Forst, 2015; Kaplan & Minton, 1994). Board seats have been traditionally regarded as the highest position in the corporate hierarchy that an employee can possibly reach during a lifetime career (Charkham, 1994). Being subordinates to the CEO, insider directors have tended to exhibit conformism, loyalty, and forbearance with respect to executives. If present in minority, independent board members were usually interlocked directors representing companies, with which a firm in question either had business relations (banks, insurance companies etc.) or in which it held an equity stake (Kishida, 1996).

A combination of rigid labor markets with complacent corporate oversight created a very stable and coherent basis of the modern industrial organization of Japanese companies. The same features also significantly reduced its innovativeness, diminished executives’ propensity to take risks, and promoted the standards of excessive frugality (Chen et al., 2020; Morikawa, 2020). The negative repercussions of the status quo have therefore, pushed regulators towards adoption of novel corporate governance mechanisms.

Empirical studies (e.g., Gao & Wagenhofer, 2021; Quagli et al., 2021) demonstrate that board independence is associated with improved operational outcomes, better executive accountability, and higher shareholder returns. In contrast, the domination of insiders on boards has been shown to promote managerial opportunism, increase the likelihood of managerial mishaps and corporate scandals (Nili, 2016). The Corporate Governance Reform (reform) in Japan promulgated in 2015 was, therefore, aimed at reinvigorating corporate supervision and shifting the focus of decision making towards prioritization of shareholder value creation.

Half-a-decade after the promulgation of the reform, empirical studies on its medium-term consequences remain scarce (Morikawa, 2020). The extant literature investigates the governance-performance nexus relying on the data from before the reform’s adoption (e.g., Aman & Nguyen, 2012; Kangand Shivdasani, 1995). The practitioners and research analysts community, however, have been quick to spot improvements in the corporate performance scorecards following the enactment of changes to the Code. The champions in adaptation to the reform’s requirements have been projected to record faster growth than the more lenient peers (Bloomberg, 2017).

The present paper attempts to quantify the broad consequences of the reform for corporate performance across a number of dimensions: (1) profitability and asset productivity; (2) shareholder value creation; (3) labor relations. To that end, we compiled a multiannual longitudinal data on 3405 listed Japanese companies observed over the period 2001–2020. Our econometric analysis reveals three key patterns, which have been shaped by the reform. First, in line with regulators’ and legislators’ expectations, firms have been actively adapting to the formal requirements formulated in the Code. The share of independent board members has been on a steady rise, while the average tenure of supervisory boards has been diminishing. Thus, we demonstrate that the reform has been widely accepted as an element of the new architecture of corporate governance in Japan. Second, the introduced changes to corporate governance practices have been associated with a significant improvement in operational performance of sampled companies. Average return on equity (ROE) and asset productivity have been on the rise. Firms have been optimizing their costs and balance sheet structures responding to the updated market expectations. As a result, corporate valuations, shareholder returns, and dividend payouts have been on the rise. The immediate outcomes of the reform have thus been a shift of executives’ focus towards prioritization of shareholder value over cohesion between diverse stakeholders. Acquisitions have been increasing as well fueled by quest for efficiency and operational optimization. While the compensation packages of managing and supervisory boards have increased, we also document a significantly stronger link between remuneration and performance in firms where the share of non-executive board members has seen a significant growth. A stronger compensation performance nexus attests to improved executives’ accountability and a significant change in the design of incentives, which now prioritize corporate bottom lines over adherence to the no-longer institutionally enforced social contract. Third, we show that the party most likely bearing the downside of the reform is labor. Following The reform’s enactment, we document a significant reduction in net employment creation and employee turnover. The number of part-time employees has grown significantly. Thus, it appears that Japanese companies have managed to boost their operational performance by optimizing their hiring practices.

The paper aims at prompting an in-depth discussion on the repercussions of the reform by pointing to its achievements as well as potential dangers. While we show that the Reform manages to reach its goals, dynamize the corporate sector, and remedy the long-term underperformance of Japanese companies in the eyes of international institutional investors, we also pinpoint the area, where regulatory changes are likely to produce side-effects. Job security has long been one of the major tenets of the social contract underlying the cohesion of the Japanese society. By substantially diluting it, the reform may engender unpredictable and potentially detrimental effects for societal dynamics. Therefore, our study shows that if ESG is to remain an important driver of shareholder value within the Japanese corporate sector, regulators and business owners should focus more on the social aspects of doing business, especially on the welfare of employees. Business leaders must consider the circumstances and goals of their organizations to determine which specific initiatives to prioritize, bearing in mind the possible downsides of taking no action. Developing a credible ESG position is crucial for Japanese business to continue to be attractive and resonant on the global stage, as it is now.

The paper contributes to the literature in the following three ways. First, it is the first study to comprehensively assess the 2015 reform along a number of dimensions including its repercussion for labor market and corporate valuations. Secondly, it contributes to the corporate governance literature by drawing on the empirical evidence from a natural experiment designed to substantially increase board independence: we demonstrate that independent directors are able to dynamize companies, improve their performance, and increase executives’ accountability. Thirdly, our research is consistent with empirical agency theory, as we find a positive relationship between the increase in proportion of independent directors and firm performance. Fourthly, we pinpoint the area where the reform is likely to produce long-term problems—the labor relations. It is likely that the positive outcomes of the reform are achieved through dilution of implicit job security guarantees, which have historically been one of the principal engines of economic growth in Japan.

The remainder of the paper is arranged as follows. First, we present a concise literature review and research questions. Then we summarize the data collection process and present our research methodology. The subsequent sections discuss key empirical findings and their implications.

## Literature review and research question

The primary transmission mechanism, through which the reform was expected to ameliorate corporate performance, was independent corporate oversight. The historically engrained corporate governance model in Japan relied on internal promotions and interlocked directors to staff supervisory boards. As a result, supervisory board members could not be qualified as independent by modern standards (Pichet, 2017).

Lack of board independence causes excessive lenience, forbearance, and loyalty with regards to the incumbent executives. It also reduces the role of supervisory boards to mediation and prevention of any disputes between key stakeholders.

The new Code adopted in 2015 stipulates that firms are obliged to appoint at least two supervisory board members meeting the formal criteria of independence. An increase in the number of independent directors is expected to raise the quality of corporate oversight and push executives towards prioritization of shareholders’ interests above those of other stakeholder groups.

The Code (principle 4.7) stipulates that independent directors are expected to perform four key functions on boards of public companies. To start with, they are supposed to perform advisory function by counseling executives on the possible changes to the corporate policies and day-to-day operations with the goal of putting firms on the path of sustainable growth and maximizing shareholder value. The latter represents a particularly important change in the architecture of corporate governance, as shareholders’ interests have not conventionally been perceived as a core of companies’ long-term objectives. Additionally, principle 4.2 of the Code prompts independent board members to encourage entrepreneurial initiatives while simultaneously providing independent advice on the viability of envisaged investment projects. Second, independent board members are expected to oversee managerial decision making, supervise, and if necessary, undertake remedial action (including replacement/involuntary departure of executives) in order to insure the fulfilment of companies’ priorities. Third, independent board members are expected to mitigate agency conflicts by precluding opportunistic behavior on the part of management. Finally, interests of minority shareholders and other stakeholders (e.g., employees) should be reflected and incorporated appropriately into the firms’ long-term strategies (Code, 2015).

The extant empirical literature suggests that a higher proportion of independent board members is associated with improved corporate performance along a number of dimensions. Independent directors have been shown to exhibit a lower degree of conformism with group thinking; they are also more likely to raise controversial issues, cast contrarian votes, and offer an outside perspective on the outstanding strategic issues (Forbes & Milliken, 1999). Motivated by reputation concerns, independent directors are also more likely to act as whistleblowers, thereby reducing the likelihood of accounting irregularities, window dressing, fraud, and managerial opportunism (Beasley, 1996). The quality of financial reports and corporate communication has also been shown to benefit from the presence of independent board members (Song & Windram, 2004). Additionally, since independent directors are recruited from a wider pool of talent, they may exhibit higher individual abilities and possess superior board-specific skills (Cavaco et al., 2017). Therefore, boards staffed with independent members appear to be better positioned to exercise both advisory and supervisory functions, thus contributing to better day-to-day management of firms (Byrd & Hickman, 1992; Hooghiemstra et al., 2019; Miletkov et al., 2017).

Independent boards also act as a mechanism of aligning the interests of managers and shareholders (Byrd & Hickman, 1992; Tihanyi et al., 2003). As advisors, independent directors have been documented to promote better decision making (Musteen et al., 2009) by managers; as supervisors, they have been shown to reduce the scale of excessive risk taking, opportunistic investments, wasteful resource allocation, and unnecessary diversification.

Among the drawbacks of supervisory boards dominated by outsiders, one should note the possibility of information deficit (Cavaco et al., 2017). In an attempt to preclude excessive control and interference with day-to-day operational decision making, executives may be inclined to withhold valuable information crucial for assessment of decisions’ viability. An impaired flow of information may thus compromise the boards’ ability to fulfill both their supervisory and advisory functions (Baker & Anderson, 2010; Ferreira & Adams, 2007). Directors recruited among insiders have valuable informal connections with incumbent managers and employees, which facilitate their access to material nonpublic information. The latter may be crucial in allowing the board to perform its advisory role. Therefore, some empirical papers (e.g., Coles et al., 2008; Harris & Raviv, 2008) postulate the existence of a natural point of boards’ saturation with independent members after which a lack of insiders deprives the board of material information. Career independent board members, who occupy a number of independent directorships, may also be less effective in performing their supervisory tasks because of their desire to keep board positions after subsequent reelections. The same career concerns are, however, present in case of board members recruited inside the firm (Hermalin & Weisbach, 1998).

Empirical studies concerning the nexus between the quality of corporate governance and firms’ performance in Japan yielded mixed results. It is worth noting, however, that such studies are predominantly based on data from the period preceding the introduction of the discussed corporate governance reform. For example, Aman and Nguyen (2012) documented a lack of significant relationship between the share of independent board members and operational performance of non-financial companies in Japan. Similar findings covering a different subperiod of analysis are reported by Kangand and Shivdasani (1995).

Kaplan and Minton (1994) identified a measurable negative impact of appointments of outside directors on contemporaneous operational performance. The discovered link may be attributed to information deficit faced by independent board members, who join boards dominated by insiders. It is worth noting that the study concerns the period of analysis, when supervisory boards of Japanese companies were predominantly staffed with directors recruited from inside (Bonn et al., 2004). Sakawa and Watanabel (2018) analyzed the link between the share of independent directors and the performance of financial institutions: their findings suggest that higher board independence does not contribute to improved operational outcomes. It is worth noting that the specificity of the financial sector could have partially contributed to the result.

Liu et al. (2011) were the first to underline the role of independent board members in prioritizing shareholders’ interests over those of other stakeholder groups within Japanese companies. Insider directors, who were recruited among firms’ employees, have tended to exhibit complacency with regards to managerial decisions and prioritize job security—a conventional tenet of social contract underlying Japanese industrial organization. The pre-existing system put a strong emphasis on stakeholders’ cohesion at the expense of corporate performance and shareholder wealth maximization. The increase in the share of independent board members has been shown to bring about significant shifts in this paradigm. In particular, boards dominated by independent directors have been shown to be associated with higher sensitivity of executive turnover to operational performance (Liu et al., 2011). A strong nexus between firms’ bottom lines and the likelihood of management departure or replacement is an attribute of enforcement of executive accountability. The more stringent corporate oversight and lower probability of board capture by incumbent managers are likely to stand behind the observable shift towards stronger pay-outcome link. Liu et al. (2011) also document a significantly lower proclivity to cut dividend payment and other forms of shareholder payouts among companies with higher proportion of independent board members. Thus, empirical evidence strongly suggests that the principal merit of independent boards resides in their ability to bring to the fore the interests of shareholders by tightening control over executives’ decisions and maintaining control of deployment of internally generated financial resources.

Changes implemented in the Code (2015) pursue the goal of increasing the role of supervisory boards in reducing the scale of agency conflicts of interest (Anderson et al., 2004; Beasley, 1996; Brickley et al., 1994). The new Code stipulates that firms are obliged to appoint at least two supervisory board members meeting the formal criteria of independence. Research shows that increasing the number of independent board members has a positive impact, inter alia, on reducing the likelihood of accounting irregularities, window dressing, fraud, and managerial opportunism (Beasley, 1996) and quality of financial reports (Song & Windram, 2004). Therefore, the operational performance of Japanese companies should benefit from the changes since the modernized boards are expected to prioritize shareholders’ interests over those of other stakeholders. Having five years of empirical data at hand, the present paper attempts to verify whether the reform’s implementation has been able to stand to legislators’ expectations. The first research question is therefore, formulated as follows:

### RQ1.

Did the implementation of the 2015 reform ameliorate corporate performance within Japanese public companies?

Additionally, we try to establish whether the reform was able to accentuate shareholder value maximization as a fundamental criterion of firms’ efficiency.

If the answer to RQ1 turns out to be affirmative, we further aim at establishing the channels through which Japanese companies were able to achieve superior operational performance and increase shareholder wealth. The Japanese system of corporate governance was notorious for prioritizing the interests of employees over those of shareholders (Shleifer & Vishny, 1997). In surveys, Japanese corporate managers are significantly likelier than executives from other countries to indicate that firms belong to all stakeholders rather than shareholders only, while the focus of firms’ activities should reside in maintaining job security rather than maximizing shareholder payouts**.** This might be the result of an ethos that has endured in Japan’s corporate culture. Today, many Japanese companies make commitments to their surrounding communities and wider society, promising “lifelong employment, environmentally conscious production processes, customer-oriented products and services, and product safety” (Suzuki & Tanimoto, 2005). Such priorities might be rooted in the distant past, when the Japanese companies were encouraged to think beyond profits, to care not only for the sole benefit of a business but also for the benefit of society (Boardman & Kato, 2003). However, recently, in most developed economies, irregular employment, such as part-time and temporary work, has shown a significant upward trend. Japan, which has a strong employment protection for regular workers, has witnessed a similar dynamic. The stagnant economy and competitive pressure have forced Japanese firms to increasingly regard irregular employees as a means to lower labor costs and to gain flexibility in hiring and dismissal (Kuroki, 2012). Additionally, prior research indicates that in Japan, internationalization has been progressing at the workers’ expense, with labor’s relative share in national income decreasing and the proportion of irregular workers increasing (Hirano &Yamada 2018).

Yamada & Hirano (2015) and Aoyagi and Ganelli (2015) show that corporate productivity growth in Japan has exceeded that of labor costs, thus breaching the long-term management-labor compromise. The long-term employment security incentivized employees to generate productivity gains through long-term skill formation (Yamada & Hirano, 2015). This important feature of Japan’s social contract and industrial organization appears to be the key target of the discussed corporate governance reform. In quest for improved efficiency, flexibility, and cost optimization, firms are likely to cut back on employment guarantees (Cammett & Posusney, 2010; Rubery et al., 2016) and labor-related expenses (Yamada & Hirano, 2015). In our study, we wanted to investigate whether while focusing on operational improvements, Japanese companies cut back on employment guarantees and labor-related expenses. In order to check this conjecture, we formulate the following research question:

### RQ2.

Did the reform’s adoption cause an erosion of job security within Japanese public companies?

The study aims at comprehensively assessing and quantifying the repercussions of the reform along a number of dimensions. We try to establish whether the reform managed to fulfil its formal goals, i.e., lead to a substantial increase of board independence, and whether it led to a deeper reconstruction of the architecture of corporate governance with goals and priorities of supervisory boards shifting towards shareholder value maximization. The latter has both upsides and downsides as the paper aims to exemplify. The following section presents the data which was collected to tackle the formulated research questions and summarizes the methodology utilized in the study.

## Data collection and methodology

For the purposes of the present study, we assembled a multiannual firm-level panel data covering 3405 listed Japanese companies. The dataset includes only non-financial firms since we focus on operational performance in our empirical analysis. The period covered in the study is between 2001 and 2020, thus it incorporates subperiods preceding and following the introduction of the 2015 corporate governance reform. Any firm, for which we did not manage to compile complete financial records was ejected from the sample. We constrained data selection to companies with non-zero revenues and total assets at any given moment during the analyzed period. Firm-level financial data (size, profitability, asset base, leverage, liquidity, growth, dividend payments and stock repurchases etc.) were assembled from the Thomson Reuters Database. In addition to financial reports, we collected data regarding the corporate governance settings within each company. The variables of interest used in our empirical analysis include: board size, number of independent board members, board independence (%), average board tenure, board-specific skills, total executive compensation and total board compensation. It is worth noting that corporate governance data were not available for all sampled companies due to differences in disclosure across companies. Finally, we also collected data on yearly employment within each company along with repartition into full- and part-time employees. Relying on these data, we managed to estimate yearly turnover and net employment creation, which are further used to gauge changes in firms’ employment practices after the adoption of the corporate governance reform.

The entire sample comprises 49,803 firm-year observations. The complete list of variables used in our empirical analysis is presented in Table 1. The financial data were scaled using total contemporaneous assets as deflator. All scale variables were winsorized at 1% and 99% levels to account for the possible impact of outliers. Summary statistics for the research sample are presented in Table 2.

**Table 1:** Definitions of variables

Variable	Definition
COMP.COM.IND	Independence of compensation committee measured as a percentage of independent supervisory board directors in the compensation committee
BOARD.SIZE	Number of members in a supervisory board
AVG.TENURE	Average tenure of supervisory board members
BOARD.IND	Percentage of independent members on a supervisory board
N.IND.MEM	Number of independent board members on a supervisory board
BOARD.IND.LOW.25	Binary variable encoding firms whose supervisory boards have less than 25% of independent members
BOARD.IND.25.50	Binary variable encoding firms whose supervisory boards have more than 25% but less than 50% of independent members
BOARD.IND.50.75	Binary variable encoding firms whose supervisory boards have more than 50% but less than 75% of independent members
BOARD.IND.HIGH.25	Binary variable encoding firms whose supervisory boards have moe than 75% of independent members
EX.COMP	Total senior executive compensation (log-transformed)
REFORM	Binary variable encoding firm-year observations covering the time period following the enactment of corporate governance reform of 2014
BOARD.COMP	Total compensation of supervisory board members (log-transformed)
PART.TIME.EMPL	Number of part-time employees in the company's workforce at the end of a reporting year
DIV.PAYOUT	Dividend payout ratio (total dividend paid divided by net earnings)
EV.EBITDA	Enterprise value to EBITDA multiple
ROE	Return on equity
OPM	Operating profit margin estimated as a ratio of normalized operating profit to total revenues
PRODUCTIVITY	Revenue divided by total number of equivalent full-time employees (log-transformed)
CASH	Total cash and cash equivalents scaled by total assets
TANGIBILITY	Property/plant and equipment scaled by total assets
FIRM.SIZE	Natural logarithm of firms' total assets
ASSET.TURN	Asset turnover ratio estimated as a ratio of total revenues to total assets
OCF	Operating cash flows scaled by total assets
INVESTMENT	Total investment cash outflows scaled by total assets
EXT.FINANCING	Net cash flows from financing activities scaled by total assets
D.CASH.YoY	YoY change in cash balance scaled by total assets
NET.EMPL.CREAT	Net employment creation estimated as a YoY percentage change in the total number of equivalent full-time employees
EMPL.TURNOVER	Percentage of employees who were terminated voluntarily or involuntarily during a given year and subsequently replaced by new hires
ACQUISITION	Cash outflows for purchases of equity stakes in other businesses scaled by total assets
DIVIDEND	Total cash dividend paid to shareholders scaled by total assets

**Table 2:** Descriptive statistics

Variable	Mean	SD	Min	Max
COMP.COM.IND	41.383	34.956	0	100
BOARD.SIZE	12.117	4.627	1	39
AVG.TENURE	6.639	3.449	0.5	37.35
BOARD.IND	17.386	15.918	0	100
N.IND.MEM	2.127	1.86	0	13
BOARD.IND.LOW.25	0.705	0.456	0	1
BOARD.IND.25.50	0.249	0.432	0	1
BOARD.IND.50.75	0.04	0.196	0	1
BOARD.IND.HIGH.25	0.006	0.079	0	1
EX.COMP	19.836	0.994	13.385	25.322
BOARD.COMP	12.728	1.021	5.701	17.981
PART.TIME.EMPL	1496.965	5734.67	0	186,000
DIV.PAYOUT	34.888	43.438	0	316.805
EV.EBITDA	9.105	12.458	−1.838	91.959
ROE	4.865	15.178	−77.986	44.724
OPM	6.02	10.032	−36.768	48.859
PRODUCTIVITY	17.502	0.808	15.36	19.688
CASH	0.189	0.153	0.012	0.743
TANGIBILITY	0.262	0.219	−0.357	0.897
ASSET.TURN	1.107	0.636	0.06	3.491
OCF	0.055	0.071	−0.228	0.261
INVESTMENT	0.04	0.057	−0.125	0.272
EXT.FINANCING	−0.004	0.079	−0.188	0.401
D.CASH.YoY	0.008	0.051	−0.145	0.223
NET.EMPL.CREAT	3.896	10.128	−15.706	63.258
EMPL.TURNOVER	4.711	5.185	0.172	27.57
ACQUISITION	0.018	0.039	−0.004	0.247
DIVIDEND	0.01	0.009	0	0.052

The empirical study is comprised of four stages. At stage one, we use univariate statistical tests to check for changes in financial management policies at firm level following the implementation of the 2015 Reform. The goal of this part of analysis to elucidate the empirically observable patterns in financial decision making, which were engendered by the shift in the composition of supervisory boards prompted by evolution of regulatory framework. These changes are tracked along four dimensions: (1) operational performance measured by operating profit margins and overall return on equity; (2) shareholder value creation proxied by valuation multiples and shareholder payouts; (3) acquisitions; (4) employment practices measured by employee turnover, employment creation and number of part-time employees. The changes are measured across time—i.e., we compare subperiods preceding and following the enactment of the new Code, and across firms with the share of independent board members serving as experimental variable for repartition of the research sample.

At stage two, we use multivariate econometric modeling to corroborate the findings reported at stage one. Relying on dynamic panel modeling GMM-SYS (Arellano & Bond, 1991), we attempt to establish whether the changes in the composition of supervisory boards of Japanese companies are associated with significant shifts in their operating performance and financial policies. We run a number of regression models with the following baseline specification:

1 $$ \begin{gathered} PERFORM_{it} = \beta_{0} + \beta_{1} PERFORM_{it - 1} + \beta_{2} PERFORM_{it - 2} + \beta_{3} REFORM_{i} + \beta_{4} BOARD.IND_{it} + \hfill \\ \beta_{5} FIRM.CONTROL_{it} + \beta^{\prime}Year_{i} + \beta^{\prime}Industry_{i} + \beta^{\prime}Error_{ij} , \hfill \\ \end{gathered} $$

where ${PERFORM}_{it}$—firm-level operational performance indicator (OPM, ROE etc.) or discretionary financial policy variable (DIVIDEND, CASH, ACQUISITION etc.), ${REFORM}_{i}$—binary variable encoding the subperiod following the enactment of the 2015 reform, ${BOARD.IND}_{it}$—percentage of independent supervisory board members, ${FIRM.CONTROL}_{it}$—a set of firm-level control variables. All models include lags of dependent and (whenever justified by the applied analytical framework) independent variables, year and industry fixed effects. We run a number of auxiliary statistical tests to verify the validity of baseline econometric model to insure that it may be used for statistical inference. The set of control variables includes proxies for firms’ size (SIZE), investment demand (INVESTMENTS), asset structure (TANGIBILITY) and productivity (PRODUCTIVITY).

In order to verify whether the interrelation between board independence and corporate performance indicators has nonlinearities, we create a number of additional binary variables—BOARD.IND.LOW.25, BOARD.IND.25.50, BOARD.IND.50.75, BOARD.IND.HIGH.25—in order to encode subsamples of companies with difference percentages of independent directors. The subsample of particular interest comprises firms in which independent supervisory board members constitute a majority (>50%) or a supermajority (> 75%). Thus, we attempt to verify whether the repercussions of the corporate governance reform are more pronounced within firms, which overhauled their internal corporate oversight by re-staffing the board completely with outsiders.

Stage three of our study extends the analysis of firm-level financial management policies by focusing on patterns of discretionary cash flow allocation during a fiscal year. Decisions regarding the deployment of firms’ internally generated cash flows are inherently interrelated. Therefore, their dynamics needs to be analyzed in conjunction relying on simultaneous equation methodology. We use methodology proposed by Gatchev et al. (2010), as it allows to effectively address endogeneity concerns and analyze interactions between the key experimental variables. Moreover, this methodology is often used in similar research on corporate governance (e.g. Benlemlih, 2017; Kuo, & Hung, 2012).

It models contemporaneous operational cash flow allocation among four alternative uses relying on a system of simultaneous equations:

2 $$\left\{\begin{array}{l}{INVESTMENT}_{it}={\alpha }_{1i}+{\beta }_{1}{OCF}_{it}+{\beta }^{^{\prime}}FIRM.CONTRO{L}_{it}+{\varepsilon }_{1it}\\ {DIVIDEND}_{it}={\alpha }_{2i}+{\beta }_{2}{OCF}_{it}+{\beta }^{^{\prime}}FIRM.CONTRO{L}_{it}+{\varepsilon }_{2it}\\ {-EXT.FINANCING}_{it}={\alpha }_{3i}+{\beta }_{3}{OCF}_{it}+{\beta }^{^{\prime}}FIRM.CONTRO{L}_{it}+{\varepsilon }_{3it}\\ {D.CASH.YoY}_{it}={\alpha }_{4i}+{\beta }_{4}{OCF}_{it}+{\beta }^{^{\prime}}FIRM.CONTRO{L}_{it}+{\varepsilon }_{4it}\end{array},\right.$$

where ${OCF}_{it}$—operating cash flows generated by company i during fiscal year t, ${INVESTMENT}_{it}$—total investment cash flow englobing capital expenses, ${DIVIDEND}_{it}$—total shareholder payouts in the form of cash dividends, ${EXT.FINANCING}_{it}$—cash flows directed towards repayment of debt (or cash obtained by issuance of new debt) or disbursement of equity (or cash obtained from new equity issuances), ${D.CASH.YoY}_{it}$—change in end-of-year cash balance reported by the company in its balance sheet. The proposed methodology relies on the standard cash flow statement data and assumes that all contemporaneously generated operating cash flows are consumed by the four enumerated uses. The so designed empirical test intends to uncover the intertemporal shifts in the firms’ propensity to invest, accumulate cash and pay dividends. One of the primary concerns voiced by regulators prior to the Code’s enactment was the low propensity of Japanese companies to invest accumulated cash reserves (Morikawa, 2020). The study attempts to establish whether an overhaul of the corporate governance system managed to change this pattern.

Subsequently, we check whether an increase in the percentage of independent board members contributed to strengthening executives’ accountability. To that end, we run static panel regression models of the following specification:

3 $$ COMPENSATION_{it} = \beta_{0} + \beta_{1} ROE_{it} + \beta_{5} FIRM.CONTROL_{it} + \beta^{\prime}Year_{i} + \beta^{\prime}Industry_{i} + \beta^{\prime}Error_{ij} $$

where ${COMPENSATION}_{it}$—either executive (EX.COMP) or supervisory board (BOARD.COMP) compensation during a given year (log-transformed), ${ROE}_{it}$—contemporaneous return on equity. Under a sound system of corporate oversight, executive compensation should be linked to firms’ operating performance (and shareholder returns which are correlated with return on equity). A weak performance-compensation link may attest to deficient system of corporate accountability, misalignment of management’s and shareholders’ interests or inappropriately designed mechanism of remuneration. Test (3) allows us to check whether the performance-pay link became stronger or weaker following the introduction of regulatory changes. Using static panel analysis, we quantify the mentioned link separately within each individual firm and among sampled firms in general. A sound country-wide system of corporate oversight would be a one in which: (1) better performing companies pay higher compensations to their management and supervisory boards (the ‘between’ variance in the random-effect static panel modeling); (2) within each specific firm, a better performance during a particular year is associated with higher yearly compensation (the ‘within’ variance in the random-effect static models).

At the final stage of our empirical analysis, we verify how the implementation of the Code impacted the employment practices of Japanese public companies. We run static panel models with the explained variable being net employment creation (NET.EMPL.CREAT)—a year-on-year percentage change in the total number of equivalent full-time employees. The key explanatory variable is BOARD.IND along with binary variables derived from it.

Overall, our empirical study attempts to establish whether the reform of 2015 managed to reach its goals—i.e., re-invigorate corporate oversight, improve firms’ performance, and shift the focus of strategic decision making towards maximization of shareholder value. Along the way, we check whether any negative repercussions for other stakeholders may ensue therefrom.

## Commentary to the key econometric findings

The analysis of dynamics of the key experimental variables in time allows us to draw a number of important conclusions regarding the impact of the 2015 reform on corporate oversight and financial management policies within Japanese companies.

**Fig. 1:**
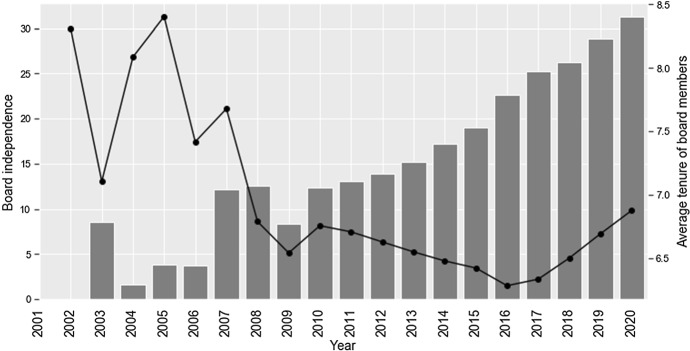
The dynamics of board independence (left axis, bar chart) and average tenure of supervisory boards members (right axis, line chart) following the enactment of corporate governance reform

To start with, in line with expectations, we document a significant increase in the average percentage of independent board members across sampled companies following the Code’s enactment (Fig. 1). The average BOARD.IND increased fourfold from ca. 8.2% in 2003 to ca. 32% in 2020. The increase has gone through three stages: (1) a gradual increase in 2003–2007; (2) plateau in 2008–2014; (3) rapid increase in 2015–2020. The updated regulatory requirements have accelerated the already ongoing but slowly progressing trend towards rendering supervisory boards more independent from executives. At the same time, average board tenure dropped from its peak value of ca. 8.4 in 2005 to less than 7 years in 2020. Supervisory boards have seemed an influx of outside directors, which was the main goal of the reform.

**Fig. 2:**
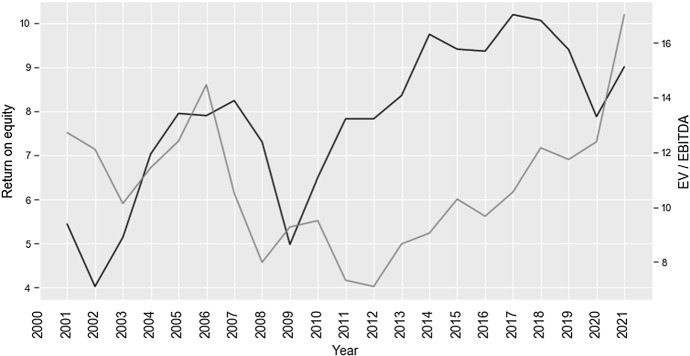
Average return on equity (left axis) of Japanese companies and EV-to-EBITDA multiples (right axis) following the enactment of reforms

Spectacular improvement is observed in corporate profitability indicators. Average ROE increased substantially following the introduced regulatory changes and reached ca. 9.4% in 2019 prior to the 2020 pandemic (Fig. 2). The frequent complaints of international institutional investors was that Japanese companies were lagging their peers from OECD in terms of key performance indicators. The disadvantage observed for a good part of the past decade appears to be gradually eroded. In response to the improvement of operating performance, valuation multiples (EV/EBITDA) of listed companies increased significantly from ca. 5.3 in 2014 (prior to Code’s enactment) to ca. 8.0 in 2020. Such a significant increase marks an important gain for firms’ shareholders and a general shift in companies’ focus towards shareholder value creation.

**Fig. 3:**
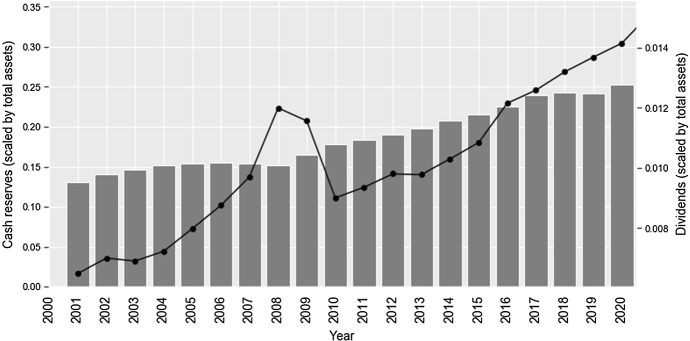
The dynamics of cash reserves (left axis, bar chart) and dividend payments (right axis, line chart)

Japanese firms continue to stash unparalleled amounts of cash reserves (Fig. 3). Average cash balanced of listed companies increased from 21% of total assets in 2014 to ca. 26% in 2020. However, this indicator is gradually plateauing with the pattern being particularly evident once the huge wave of monetary expansion of the last 2 years is taken in account. Perhaps the most striking change is a 40% increase in the relative amount of dividend payouts during the period between 2014 and 2020 (average dividend payments increased from ca. 0.1% of total assets to 0.14% in 2020). The expansion of dividends is another sign of the corporate focus shifting towards prioritization of shareholders’ interests.

The results of univariate statistical analysis comparing subperiods preceding and following reform’s implementation corroborate our observations (Table 3). Student’s t-test for the difference of cross-sample means shows that BOARD.IND increased from 11.26% to 23.14% (sig.: 1%). The average share of independent directors sitting on compensation committees increased from 37.32% to 45.46% (sig.: 1%). ROE increased almost twofold from 3.45% to 7.09% (sig.: 1%). Increases of both operating profit margin (from 5.59% to 6.69%, sig.: 1%) and asset turnover (from 1.10 to 1.11, sig.: 1%) have contributed to the improved returns. Leverage also saw a significant increase: prior to the reform, an average company was deleveraging at the pace of 0.9% of total assets per year. After the enactment of the reform, the corporate sector started incurring new debts at the rate of 0.3% of total assets per year. Board and executive compensations increased (sig.: 5%). Japanese companies have also become much more acquisitive with the average relative size of M&A deals increasing from 0.015 to 0.023 (sig.: 1%).

**Table 3:** Cross-sample differences of key corporate policies before and after reform enactment

	Mean1	Mean2	dif	St_Err	t_value	*p*_value
*A. Changes in the corporate governance practices*						
AVG.TENURE	6.825	6.472	0.353	0.107	3.3***	0.001
BOARD.IND	11.258	23.14	−11.882	0.452	−26.3***	0
N.IND.MEM	1.342	2.864	−1.522	0.052	−29.35***	0
COMP.COM.IND	37.323	45.458	−8.135	2.089	−3.9***	0
EX.COMP	19.746	19.887	−0.142	0.075	−1.9*	0.059
BOARD.COMP	12.623	12.801	−0.178	0.057	−3.15***	0.002
*B. Changes in firm financials*						
ROE	3.454	7.098	−3.643	0.135	−26.95***	0
OPM	5.594	6.688	−1.093	0.089	−12.25***	0
ASSET.TURN	1.101	1.117	−0.017	0.005	−2.9***	0.004
INVESTMENT	0.038	0.042	−0.004	0.001	−8.15***	0
D.CASH.YoY	0.003	0.015	−0.011	0.001	−9.65***	0
EXT.FINANCING	−0.009	0.003	−0.013	0.001	−17.85***	0
CASH	0.164	0.229	−0.066	0.002	−49.25***	0
DIVIDEND	0.009	0.012	−0.003	0	−34.35***	0
EV.EBITDA	8.883	9.463	−0.581	0.116	−5***	0
*C. Changes in employment practices*						
PRODUCTIVITY	17.495	17.512	−0.017	0.007	−2.2**	0.028
NET.EMPL.CREAT	4.128	3.564	0.565	0.282	2**	0.045
PART.TIME.EMPL	1457.821	3118.216	−1660.4	411.026	−4.05***	0
*D. Firms' acquisitiveness*						
ACQUISISITION	0.015	0.023	−0.009	0.001	−8.55***	0

The table presents the results of Student t-tests of differences in mean values of a number of experimental variables. Subsample (2) comprises firm-year observations following the enactment of the corporate governance reform, subsample (1) comprises firm-year observations covering the time span before reform enactment. *, ** and *** denote statistical significance of the test at 10%, 5% and 1% levels respectively

**Fig. 4:**
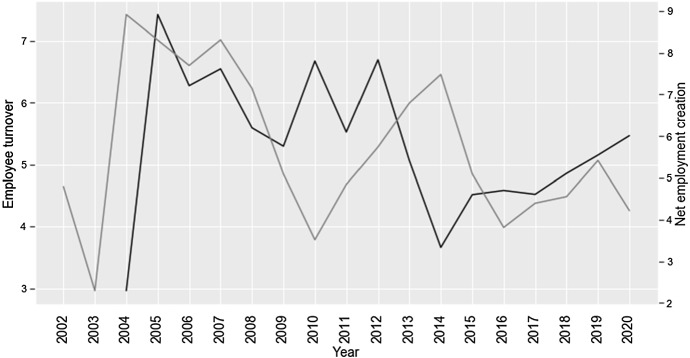
Employee turnover (left axis) and net employment creation (right axis)

In line with our conjecture formulated in the theoretical part of the paper, we find that net employment creation diminished from an average of 4.128% before to 3.56% after the Code’s enactment. The downward trend is also evident on graphic analysis (Fig. 4). The graph shows that net employment creation increased between 2010 and 2014 and began to decline after 2014. This decrease can be linked to companies’ gradual adaptation to the new Code, whose initial draft was released in 2014 (Financial Services Agency, 2014). A reduction in employment creation is accompanied with a slump in employee turnover. Firms cut back on hiring, while employees became more reluctant to switch workplace. The average number of part-time employees per company increased from 1457 before the reform to 3118 after (sig.: 1%). Thus, improvements in corporate bottom lines, expense structures and productivity appear to be partially explained with the tightening of employment creation and optimization of workforce deployment.

In order to verify, whether the discovered shifts in corporate financial policies were caused by the reform rather than other external factors, we check the impact of the key variable that the reform targeted—BOARD.IND—on the corporate financials discussed above. Table 4 presents the results of cross-sample univariate tests of differences of means with the sample having been partitioned relying on BOARD.IND as discriminatory variable. Our results unequivocally demonstrate that board independence is robustly positively associated with corporate performance and shareholder payouts. The average ROE of the bottom tercile of companies with the lowest board independence is ca. 0.36 percentage points lower than that of the middle tercile. The average ROE among the top tercile of firms is 1.327 percentage points higher than that of the middle tercile. We thus observe a monotonic increase of performance indicators along with BOARD.IND. Similar patterns may be observed in case of other operating performance metrics (e.g., operating profit margin and asset turnover). Dividend payouts are also significantly different across the three terciles: the average dividend payout is 0.14 percentage points higher in the second tercile compared to the first, and 0.36 percentage points higher in the third tercile than in the second (sig.: 1%). The same pattern is characteristic of the average size of acquisitions performed by sampled companies: firms with a higher percentage of independent directors on board are significantly more acquisitive.

**Table 4:** Corporate financials contingent upon the degree of independence of supervisory boards

	Contrast	Std.Err	t	*P* > t	[95%_Conf	Interval]
Return on equity ROE						
2_vs_1	0.365727	0.396227	0.92	0.626	−0.563241	1.294695
3_vs_1	1.692857	0.401229	4.22	0	0.7521617	2.633552
3_vs_2	1.32713	0.400954	3.31	0.003	0.3870796	2.26718
Dividend payments (scaled by total assets) DIVIDEND						
2_vs_1	0.001422	0.000402	3.54	0.001	0.0004793	0.002366
3_vs_1	0.004991	0.000407	12.26	0	0.0040367	0.005946
3_vs_2	0.003569	0.000408	8.75	0	0.0026132	0.004525
Size of corporate acquisitions (scaled by total assets) ACQUISITION						
2_vs_1	0.008764	0.003404	2.57	0.028	0.0007577	0.01677
3_vs_1	0.007058	0.003148	2.24	0.066	−0.000347	0.014463
3_vs_2	−0.00171	0.002894	−0.59	0.826	−0.008513	0.0051
Number of part-time employees PART.TIME.EMPL						
2_vs_1	596.8585	1708.305	0.35	0.935	−3419.614	4613.331
3_vs_1	5584.203	1727.045	3.23	0.004	1523.669	9644.738
3_vs_2	4987.345	2076.881	2.4	0.044	104.2973	9870.393

The table presents pairwise comparisons of mean values of three experimental variables—ROE, DIVIDEND, and ACQUISITION. Subsamples labelled 1, 2, and 3 are created by dividing the sample into three equal subsamples (terciles) relying on the distribution of the variable BOARD.IND. Subsample labelled 1 encodes 33% of firm-year observations with the lowest supervisory board independence. The table reports the results of t-tests, the respective *p*-values, and 95% confidence intervals

Interestingly, we also observe a positive associative link between board independence and the number of part-time employees reported by firms. The average number of part-timers is higher by 4987 in firms from the top tercile of the BOARD.IND distribution than in the middle tercile and 5584 higher than in the bottom tercile.

Our findings are corroborated with dynamic GMM-SYS panel modeling with fixed year and industry effects. Table 5 studies the relationship between board independence and firm-level ROE. In line with prior results, we find that the average ROE increased by 3.288 percentage points following the reform’s enactment (the respective regression coefficient is significant at 1% level). An increase of board independence by 1 percentage point is associated with an increase of ROE by ca. 0.026%. The highest ROE is recorded among firms where independent directors represent more than 75% of the supervisory boards (BOARD.IND.HIGH.25): it is higher than the sample average by ca. 5.52 percentage points (sig.: 1%).

**Table 5:** The relationship between reform enactment, board independence, and corporate profitability

Model no.	(1)	(2)	(3)	(4)	(5)	(6)
*Explained variable: return on equity (ROE)*						
L.ROE	0.288***	0.358***	0.358***	0.358***	0.357***	0.357***
	(17.23)	(7.29)	(7.29)	(7.27)	(7.25)	(7.24)
L2.ROE	0.013	0.029	0.029	0.029	0.028	0.028
	(0.91)	(0.64)	(0.64)	(0.63)	(0.62)	(0.62)
REFORM	3.288***					
	(4.61)					
BOARD.IND		0.026*				
		(1.77)				
BOARD.IND.LOW.25			−0.811			
			(−1.53)			
BOARD.IND.25.50				0.630		
				(1.17)		
BOARD.IND.50.75					0.033	
					(0.04)	
BOARD.IND.HIGH.25						5.523***
						(3.28)
Year Fixed Effects	Yes	Yes	Yes	Yes	Yes	Yes
Firm-Level Controls	Yes	Yes	Yes	Yes	Yes	Yes
Constant Term	Yes	Yes	Yes	Yes	Yes	Yes
ar1	−20.903***	−5.637***	−5.629***	−5.623***	−5.628***	−5.628***
ar2	−0.758	−1.834	−1.832	−1.830	−1.842	−1.837
chi2	1746.805***	562.537***	2984.596***	2978.659***	563.177***	576.606***

The table presents the results of dynamic panel regression modeling (GMM-SYS). All models include the first and second lag of explained variables, year fixed effects, and firm-level controls. The coefficients for some of the control variables (FIRM.SIZE, L.TANGIBILITY, L.INVESTMENTS, L.PRODUCTIVITY) and constant term are not reported for reasons of brevity. Prefixes L. and L2. indicate the first and second lag of explanatory variables respectively. Statistical significance of variables at 1%, 5% and 10% level is denoted with ***, ** and * respectively

Similar patterns are reported for the nexus between operating profit margins (OPM) and board independence (Table 6). Following the introduction of the reform, the average OPM increased by ca. 0.58 percentage points. The coefficient at BOARD.IND variable is positive and statistically significant at 5% level pointing to the positive link between OPM and BOARD.IND. The highest OPM is documented among firms with the share of independent directors exceeding 75%: the average for this subsample is higher by 3.618 percentage points than the average for the entire sample. The bottom quartile of the BOARD.IND distribution is characterized with the lowest OPM (−0.81%, sig.: 5%).

**Table 6:** The dynamics of operating profit margins following reforms’ enactment

Model no.	(1)	(2)	(3)	(4)	(5)	(6)
*Explained variable: operating profit margin (OPM)*						
L.OPM	0.587***	0.515***	0.516***	0.517***	0.514***	0.516***
	(32.86)	(7.07)	(7.11)	(7.12)	(7.09)	(7.10)
L2.OPM	0.029*	0.034	0.033	0.034	0.034	0.033
	(1.79)	(1.05)	(1.03)	(1.04)	(1.07)	(1.01)
REFORM	0.581***					
	(3.58)					
BOARD.IND		0.033**				
		(2.25)				
BOARD.IND.LOW.25			−0.813**			
			(−2.28)			
BOARD.IND.25.50				0.345		
				(1.25)		
BOARD.IND.50.75					1.533*	
					(1.78)	
BOARD.IND.HIGH.25						3.618**
						(2.49)
Year fixed effects	Yes	Yes	Yes	Yes	Yes	Yes
Firm-level controls	Yes	Yes	Yes	Yes	Yes	Yes
Constant	Yes	Yes	Yes	Yes	Yes	Yes
ar1	−16.039***	−4.041***	−4.041***	−4.036***	−4.054***	−4.027***
ar2	−0.311	−0.489	−0.503	−0.494	−0.479	−0.474
chi2	4434.744***	864.799***	2958.711***	855.901***	3038.281***	2878.807***

The table presents the results of dynamic panel regression modeling (GMM-SYS). All models include the first and second lag of explained variables, year fixed effects, and firm-level controls. The coefficients for some of the control variables (FIRM.SIZE, L.TANGIBILITY, L.INVESTMENTS, L.CASH) and constant term are not reported for reasons of brevity. Prefixes L. and L2. indicate the first and second lag of explanatory variables respectively. Statistical significance of variables at 1%, 5% and 10% level is denoted with ***, ** and * respectively

The results presented in Tables 4, 5, and 6 are consistent with the previous findings showing that a higher proportion of independent supervisory board members is associated with an improved corporate performance. By virtue of being more likely to raise controversial issues, cast contrarian votes, and offer an outside perspective on the outstanding strategic issues, independent directors appear to indirectly improve business’ bottom lines (Forbes & Milliken, 1999). Moreover, independent supervisory board members might perform both advisory and supervisory function better than insiders (Byrd & Hickman, 1992; Miletkov et al., 2017; Hooghiemstra et al., 2019), as they might exhibit superior board-specific skills (Cavaco et al., 2017).

Analysis of results if simultaneous Eqs. (2) presented in Table 7 suggests that post 2015 Japanese companies have changed the patterns of cash flow allocation significantly. In particular, in line with Morikawa (2020), we find that increasing board independence did not encourage firms to allocate more cash towards tangible investments. Before 2015, an average company directed 5.3% of the value of total assets towards tangible investments. After 2015, the share of capital expenses dropped to 2.7%, which is in line with findings reported by Morikawa (2020). At the same time, the middle and top tercile of firms in terms of BOARD.IND exhibit an above-average proclivity to invest (5.0% vs 2.7% for the entire sample). Thus, we find that board independence is positively associated with firms’ propensity to invest.

**Table 7:** The impact of corporate governance reforms on intertemporal patterns of cash flow allocation

Model no.	(1)		(2)		(3)		(4)
*A. Proclivity to invest*							
Explained variable: investment outlays scaled by total assets (INVESTMENT)							
	Complete sample		Before reform		After reform		Terciles 2 and 3*
OCF	0.050***		0.053***		0.027***		0.050***
	(11.97)		(9.41)		(3.82)		(11.84)
L.OCF	0.070***		0.060***		0.058***		0.070***
	(17.08)		(10.91)		(8.15)		(16.66)
L2.OCF	0.058***		0.054***		0.033***		0.057***
	(14.21)		(9.86)		(4.81)		(13.92)
_cons	−0.253***		−0.362***		−0.711***		−0.256***
	(−12.42)		(−11.01)		(−13.60)		(−12.38)
Year fixed effects	Yes		Yes		Yes		Yes
Firm fixed effects	Yes		Yes		Yes		Yes
Firm-level controls	Yes		Yes		Yes		Yes
Wald Chi2	63.96***		57.91***		34.89***		61.61***
*B. Proclivity to stash cash*							
Explained variable: YoY change of cash balance scaled by total assets							
	Complete sample		Before reform		After reform		Terciles 2 and 3*
OCF	0.372***		0.358***		0.439***		0.371***
	(25.81)		(17.19)		(15.57)		(24.48)
L.OCF	−0.074***		−0.091***		−0.010		−0.067***
	(−5.27)		(−4.47)		(−0.35)		(−4.55)
L2.OCF	−0.040***		−0.061***		0.034		−0.040***
	(−2.81)		(−2.85)		(1.23)		(−2.70)
_cons	−0.143**		−0.441***		−0.231		−0.137*
	(−2.08)		(−3.80)		(−1.17)		(−1.90)
Year fixed effects	Yes		Yes		Yes		Yes
Firm fixed effects	Yes		Yes		Yes		Yes
Firm-level controls	Yes		Yes		Yes		Yes
Wald Chi2	37.17***		24.31***		29.72***		33.68***
*C. Proclivity to pay dividends*							
Explained variable: cash dividends scaled by total assets							
	Complete sample		Before reform		After reform		Terciles 2 and 3*
OCF	0.007***		0.003***		0.005***		0.007***
	(14.54)		(6.23)		(6.61)		(14.13)
L.OCF	0.018***		0.013***		0.017***		0.017***
	(37.19)		(23.19)		(23.71)		(36.47)
L2.OCF	0.014***		0.010***		0.012***		0.014***
	(30.34)		(18.59)		(16.98)		(29.92)
_cons	−0.034***		−0.044***		0.017**		−0.034***
	(−13.33)		(−12.22)		(2.90)		(−13.24)
Year fixed effects	Yes		Yes		Yes		Yes
Firm fixed effects	Yes		Yes		Yes		Yes
Firm-level controls	Yes		Yes		Yes		Yes
Wald Chi2	330.51***		210.56***		197.44***		313.35***

The table presents the results of static panel regression modeling. All models include year fixed effects, firm fixed effects, and firm-level controls. The coefficients for some of the control variables are not reported for reasons of brevity. Prefixes L. and L2. indicate the first and second lag of explanatory variables respectively. Statistical significance of variables at 1%, 5% and 10% level is denoted with ***, ** and * respectively

*Terciles 2 and 3 represent the top 2 terciles of the research sample in terms of supervisory board independence

The propensity to save cash from contemporaneous cash flows has seen a significant increase post 2015. Before 2015, firms saved on average 35.8% of the value of total assets in cash and cash equivalents. After reform’s enactment, that percentage increased to 43.9%. The middle and top tercile of the BOARD.IND distribution are, however, once again diverging from the sample average (37%). These findings are also corroborated by multivariate dynamic panel modeling (Table 8). Within multivariate settings, post-reform cash reserves increased by 1.6 percentage points (sig.: 1%). The highest incremental increase is noted among firms with the share of independent directors exceeding 75–2.1% percentage points (sig.: 1%).

**Table 8:** Cash savings of Japanese companies

Model no.	(1)	(2)	(3)	(4)	(5)	(6)
*Explained variable: cash and cash equivalents scaled by total assets*						
L.CASH	0.683***	0.818***	0.818***	0.818***	0.817***	0.815***
	(43.98)	(7.55)	(7.53)	(7.52)	(7.58)	(7.46)
L2.CASH	0.047***	0.121	0.121	0.121	0.120	0.120
	(3.70)	(1.64)	(1.65)	(1.65)	(1.63)	(1.67)
REFORM	0.016***					
	(8.78)					
BOARD.IND		0.000				
		(0.18)				
BOARD.IND.LOW.25			0.000			
			(0.20)			
BOARD.IND.25.50				−0.001		
				(−0.34)		
BOARD.IND.50.75					−0.003	
					(−0.59)	
BOARD.IND.HIGH.25						0.021***
						(2.63)
Year fixed effects	Yes	Yes	Yes	Yes	Yes	Yes
Firm-level controls	Yes	Yes	Yes	Yes	Yes	Yes
Constant term	Yes	Yes	Yes	Yes	Yes	Yes
ar1	−25.126***	−4.272***	−4.268***	−4.271***	−4.255***	−4.270***
ar2	−2.803	−1.337	−1.340	−1.341	−1.337	−1.344
chi2	31,151.875***	58,371.327***	56,986.571***	57,312.447***	56,762.066***	57,531.639***

The table presents the results of dynamic panel regression modeling (GMM-SYS). All models include the first and second lag of explained variables, year fixed effects, and firm-level controls. The coefficients for some of the control variables (FIRM.SIZE, L.TANGIBILITY, L.INVESTMENTS, L.OPM) and constant term are not reported for reasons of brevity. Prefixes L. and L2. indicate the first and second lag of explanatory variables respectively. Statistical significance of variables at 1%, 5% and 10% level is denoted with ***, ** and * respectively

Finally, firms with higher percentage of independent directors are evidenced to allocate a much higher share of contemporaneous operating cash flows towards dividend payments: 0.7% against 0.5% for the remainder of the sample. As noted before, all firms significantly increased dividend payouts compared to 2015. Dynamic panel modeling summarized in Table 9 demonstrates that after 2015, average dividend payouts scaled by total assets increased by ca. 0.1 percentage points (sig.: 1%).

**Table 9:** Dividend payouts

Model no.	(1)	(2)	(3)	(4)	(5)	(6)
*Explained variable: cash dividends scaled by total assets*						
L.DIVIDEND	0.679***	0.714***	0.714***	0.714***	0.714***	0.717***
	(42.98)	(15.44)	(15.37)	(15.35)	(15.31)	(15.70)
L2.DIVIDEND	0.126***	0.072	0.071	0.072	0.072	0.072
	(7.47)	(1.75)	(1.74)	(1.76)	(1.77)	(1.76)
REFORM	0.001***					
	(8.84)					
BOARD.IND		0.000				
		(1.15)				
BOARD.IND.LOW.25			−0.000*			
			(−2.25)			
BOARD.IND.25.50				0.000		
				(1.74)		
BOARD.IND.50.75					0.000	
					(0.18)	
BOARD.IND.HIGH.25						0.003
						(1.55)
Year fixed effects	Yes	Yes	Yes	Yes	Yes	Yes
Firm-level controls	Yes	Yes	Yes	Yes	Yes	Yes
Constant term	Yes	Yes	Yes	Yes	Yes	Yes
ar1	−19.773***	−6.040***	−6.054***	−6.058***	−6.036***	−6.028***
ar2	−0.238	−0.453	−0.463	−0.462	−0.445	−0.459
chi2	16,574.461***	24,645.369***	24,862.699***	4401.566***	24,386.988***	4946.340***

The table presents the results of dynamic panel regression modeling (GMM-SYS). All models include the first and second lag of explained variables, year fixed effects, and firm-level controls. The coefficients for some of the control variables (FIRM.SIZE, L.TANGIBILITY, L.INVESTMENTS, L.OPM) and constant term are not reported for reasons of brevity. Prefixes L. and L2. indicate the first and second lag of explanatory variables respectively. Statistical significance of variables at 1%, 5% and 10% level is denoted with ***, ** and * respectively

Table 10 summarizes the tests of pay-performance relationship where performance is measured with ROE. We show that prior to the reform the strength of the ROE-executive pay relationship equaled 0.005 (sig.: 1%). After 2015, the value of the coefficient increases more than twofold to 0.012 (sig.: 1%). Thus, the implementation of the corporate governance reform appears to have increased managerial accountability, which is consistent with prior studies, which show that board independence is associated with improved operational outcomes and lower agency costs (e.g., Gao & Wagenhofer, 2021; Quagli et al., 2021).

**Table 10:** Pay-performance relationship

Model no.	(1)	(2)	(3)	(4)	(5)	(6)
*Explained variable: total executive compensation (log-transformed)*						
	Complete sample	Before reform	After reform	Terciles 1	Terciles 2	Terciles 3
ROE	0.010***	0.005*	0.012***	0.001	0.011	0.011***
	(4.419)	(1.801)	(3.966)	(0.180)	(1.835)	(4.354)
_cons	12.284***	11.460***	12.460***	16.363***	0.000	12.313***
	(10.397)	(6.393)	(10.644)	(3.423)	(.)	(11.450)
Year fixed effects	Yes	Yes	Yes	Yes	Yes	Yes
Firm-level controls	Yes	Yes	Yes	Yes	Yes	Yes
R2	0.27	0.26	0.24	0.46	0.30	0.33

The table presents the results of static panel regression modeling. All models include year fixed effects, and firm-level controls. The coefficients for control variables are not reported for reasons of brevity. Prefixes L. and L2. indicate the first and second lag of explanatory variables respectively. Statistical significance of variables at 1%, 5% and 10% level is denoted with ***, ** and * respectively

**Fig. 5:**
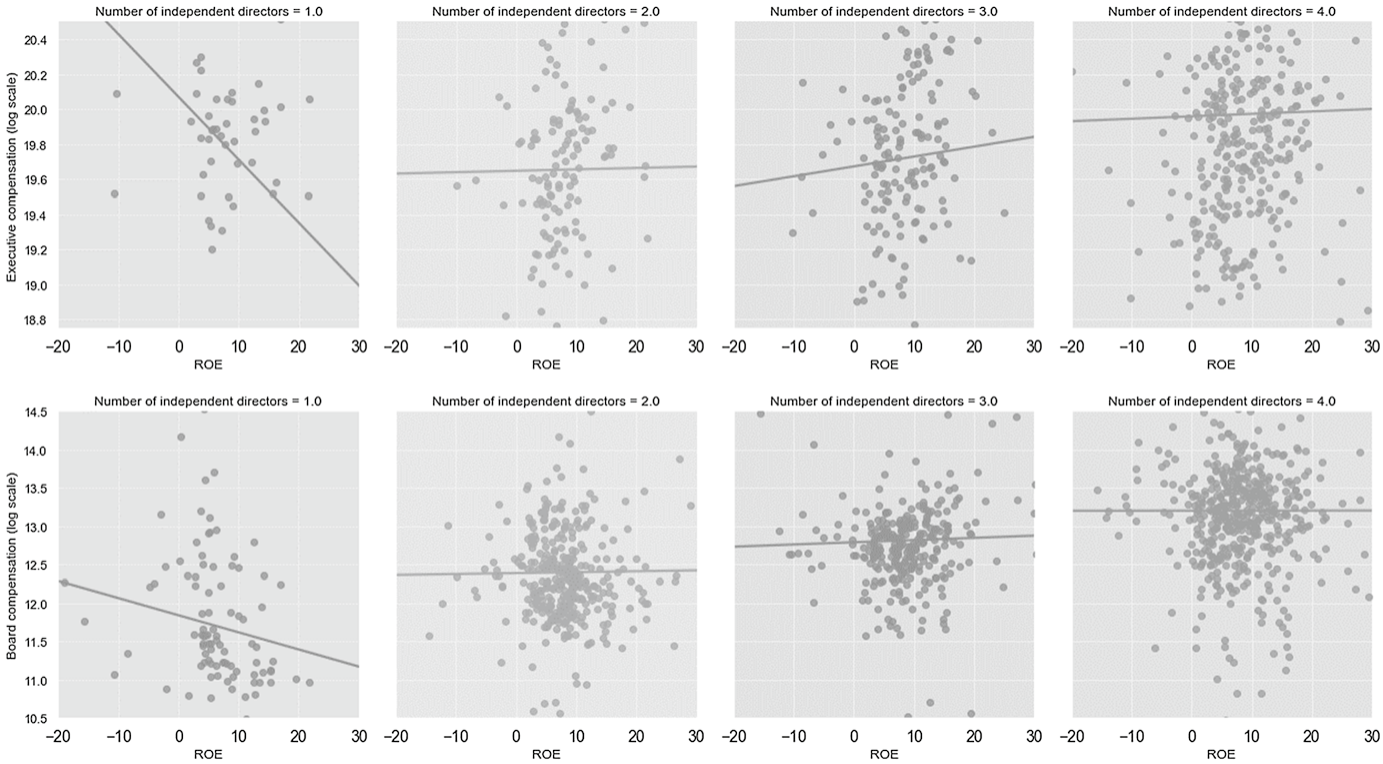
Sensitivity of executive and supervisory board compensation (log-transformed) to average ROE depending on the degree of board independence

The relationship is particularly strong in the top tercile of BOARD.IND’s distribution. Graphical analysis (Fig. 5) clearly demonstrates that board independence plays a crucial role in intermediating the pay-performance relationship. The link is negative in firms employing one or zero independent directors and becomes positive after adding more independent directors on the supervisory board.

Finally, results reported in Table 11 demonstrate that reform’s implementation caused a significant reduction in net employment creation by listed Japanese companies (−0.628 percentage points; sig.: 5%). Board independence is negatively associated with employment growth. Our findings indicate that the largest drop in net employment creation is observed in the top tercile of firms with the highest percentage of independent board members, where we also documented the largest improvement in operating performance. Research shows that in the past, in response to stagnant economy, Japanese firms have increasingly employed irregular employees to slash labor costs and to gain flexibility in hiring and dismissal (Kuroki, 2012). Therefore, in quest for improved efficiency, flexibility, and cost optimization, Japanese companies might be likely to cut back on employment guarantees (Cammett & Posusney, 2010; Rubery et al., 2016) and labor-related expenses (Yamada & Hirano, 2015). Our findings suggest that performance gains may at least in part be explained by the more conservative hiring policies adopted by Japanese companies after 2015.

**Table 11:** Net employment creation contingent upon board independence

Model no.	(1)	(2)	(3)	(4)
*Explained variable: net employment creation*				
**REFORM**	−0.628**			
	**(**−**2.326)**			
**BOARD.IND**		−0.021*		
		**(**−**1.851)**		
**BOARD.IND.LOW.25**			**0.623**	
			**(1.616)**	
**NINDMEM**				−0.173*
				**(**−**1.781)**
_cons	17.598***	16.300***	16.032***	15.271***
	(3.260)	(2.965)	(2.915)	(2.719)
Year fixed effects	Yes	Yes	Yes	Yes
Firm-level controls	Yes	Yes	Yes	Yes
Wald Chi2	125.18***	90.08***	89.61***	89.59***

The table presents the results of static panel regression modeling. All models include year fixed effects, and firm-level controls. The coefficients for some of the control variables are not reported for reasons of brevity. Prefixes L. and L2. indicate the first and second lag of explanatory variables respectively. Statistical significance of variables at 1%, 5% and 10% level is denoted with ***, ** and * respectively

## Concluding remarks

The main objective of the Japan Corporate Governance Code was to contribute to the broader Japan Revitalization Strategy (Spiegel, 2018) by intensifying pressure on company executives to implement operational restructuring and to increase business efficiency. One of the key interim purposes was to increase the independence of supervisory boards. Updated in 2021, the Code identified ESG as one of the key pillars of well-functioning organizations.

Our study shows that increasing the participation of independent members in supervisory boards has had a positive impact on the performance of Japanese companies measured by corporate profitability, asset productivity, dividend payouts, acquisitions’ value, and valuation multiples. We also document a significant increase in sensitivity of executives’ and directors’ compensations to the dynamics of firms’ bottom lines. These findings accord with the fundamental tenets of agency theory (Fama & Jensen, 1983; Jensen & Meckling, 1976), which posits that supervisory board independence strengthens the effectiveness of board monitoring which, in turn, ameliorates firms’ internal business processes.

While drawing attention to the positive consequences of the reform, we also underline the possible problems. In particular, we pinpoints a decline in net employment creation and employee turnover accompanied with a significant increase in the number of part-time employees across firms, which experienced post-reform improvements in operating performance. While the reform managed to boost firms’ competitiveness, it also appears to undermine the preexisting social contract, which emphasizes job security.

This effect should be a red light for regulators and business owners as prior research shows that a more employee-oriented management strategy can have a discernable positive impact on a company’s performance. The extant literature also suggests that employee satisfaction is positively correlated with shareholder returns (Edmans, 2011). Additionally, geography-specific empirical studies show that Japanese workers prioritize employment security in exchange for bringing about productivity gains through long-term skill formation, which appears to be a reliable predictor of firms’ long-term operating performance (Yamada & Hirano, 2015).

Due to unavailability of relevant employee-level data, which we indicate as a limitation of this study, we are unable to pinpoint the detailed cross-sectional characteristics of the identified reduction in net employment creation and employee turnover. Therefore, we are unable to ascertain whether the documented changes have occurred only in labor contracts of employees continuing to work full-time or whether the trend affects all employees uniformly. We identify these problems as important directions for future research. Additional survey data would help elucidate whether an increase in irregular employment is perceived as a major issue by employees of the Japanese corporate sector and whether it may negatively impact their productivity and workplace morale.

A careful examination of changes caused by the new Code is relevant from the standpoint of business executives and regulators alike. The initial results broadly suggest an overall beneficial impact of the reform on the corporate sector’s performance, but a more detailed analysis reveals that some of the fundamental tenets of Japan’s corporate culture may have been eroded as a result.
